# Associations between ultra-processed food consumption and duration of exercise with psychological symptoms in Chinese adolescents: a nationwide cross-sectional survey

**DOI:** 10.3389/fnut.2025.1591909

**Published:** 2025-06-17

**Authors:** Wei Zheng, Jianping Xiong, Bo Huang, Qingtao Kong

**Affiliations:** ^1^School of Physical Education and Sport Science, Fujian Normal University, Fuzhou, Fujian, China; ^2^School of Physical Education, Jiangxi University of Finance and Economics, Nanchang, Jiangxi, China; ^3^School of Physical Education, Shangrao Normal University, Shangrao, Jiangxi, China; ^4^Department of Physical Education, Shanghai Ocean University, Shanghai, China

**Keywords:** ultra-processed food consumption, duration of exercise, psychological symptoms, adolescents, China

## Abstract

**Background:**

Globally, ultra-processed food (UPF) consumption among adolescents is increasing, and the duration of exercise is decreasing, which has a serious negative impact on adolescents’ physical and mental health. In addition, the prevalence of psychological symptoms among Chinese adolescents is also increasing, which affects adolescents’ lives and academic performance. However, the association between UPF consumption, duration of exercise, and psychological symptoms among Chinese adolescents is still unclear. This study aims to analyze the association between UPF consumption, exercise duration, and psychological symptoms in Chinese adolescents.

**Methods:**

This study used cross-sectional data from 2023 on 14,445 adolescents aged 13–18 years in five regions of China. In this study, a self-assessment questionnaire was used to assess UPF consumption and duration of exercise, and the Brief Instrument on Psychological Health of Youths (BIOPHY) questionnaire was used to assess adolescents’ psychological symptoms. In addition, factors affecting participants’ psychological symptoms were evaluated for height, weight, grip strength, standing long jump, father’s education, mother’s education, commuting style, and sleep duration. The t-test, chi-square test, binary logistic regression analysis, and ordered logistic regression analysis in a generalized linear model were used to assess the existence of associations between UPF consumption, duration of exercise with psychological symptoms.

**Results:**

The prevalence rates of emotional problems, behavioral problems, and social adjustment difficulties among Chinese adolescents were 28.2, 28.0, and 17.6%, respectively; and the prevalence rate of psychological symptoms among Chinese adolescents was 22.0%. Adolescents with lower exercise durations (<30 min/day) and higher UPF consumption (>6 times/week) were 1.54 times more likely to experience psychological symptoms compared to their peers with healthier behaviors.

**Conclusion:**

Ultra-processed food consumption and duration of exercise were associated with psychological symptoms in Chinese adolescents. The higher the UPF consumption and the shorter the duration of exercise, the higher the prevalence of psychological symptoms. These findings highlight the need for public health strategies focusing on reducing UPF consumption and promoting regular exercise to mitigate psychological symptoms among adolescents.

## 1 Introduction

According to the World Health Organization (WHO) in 2021, 1 in 7 people aged 10–19 years suffer from psychosomatic symptoms, which account for 13% of the global burden of disease in this age group (1). Data results from the Global Burden of Disease (GBD) 2019 study show that there are a total of 2.516 billion adolescents between the ages of 5 and 24 years old globally, of which 293 million suffer from at least one psychological symptom, with an average prevalence rate of 11.63% (2, 3). Similarly, China, a developing country, is no exception. Research confirms that an epidemiological survey in China in 2022 found that the prevalence of psychological symptoms among adolescents aged 6 to 16 years was 17.5% and that boys had a higher prevalence of psychological symptoms than girls, while the prevalence of depression and anxiety disorders was higher among girls, and the prevalence of behavioral disorders was higher among boys (4).

However, factors affecting adolescent health are multifaceted and include the physical effects of diet and physical activity. A survey of nutritional and healthy lifestyles among adolescents in Europe showed that adolescent dietary behaviors are an important basis for population health and encouraged adolescents to eat sensibly (5). Another survey of adolescents in the North African country of Morocco showed a strong correlation between the nutritional intake status of adolescents and physical fitness and body composition, and encouraged adolescents to maintain a balanced diet and be physically active, which is essential (6). This shows the positive impact of sensible eating behaviors in adolescence on future health in adulthood. Another study of Asian adolescents also showed the positive impact of sensible dietary behaviors and physical activity on adolescent health, and advocated sensible diet and physical activity for adolescents (7).

Studies have confirmed the association between adolescent psychological symptoms and obesity, cardiovascular disease, and type 2 diabetes, which are also widely recognized to be associated with overeating and insufficient exercise (8, 9). Studies have found that Western diets rich in fast food and added sugar are positively associated with psychological symptoms (10). As defined by the NOVA Food Classification System, UPF is an energy-dense, ready-to-eat, industrially formulated food (11, 12). A meta-analysis showed that suggests that dietary patterns of low UPF intake may have a wide range of public health benefits (13). It has also been shown that a 10% increase in the intake of ultra-processed foods is associated with a 12% increase in the overall risk of cancer and an 11% increase in the risk of breast cancer; conversely, for every 10% increase in the intake of unprocessed foods (NOVA Group 1 food) in the diet, there will likely be a reduction in the overall cancer risk (14). China is no exception, and the China Nutrition Survey shows that UPF consumption among adolescents is on the rise year after year, negatively affecting adolescents’ physical and mental health (15). However, the association between UPF consumption, the highest level of food processing according to the NOVA classification, and adolescent psychological symptoms is unclear.

Surveys show that physical activity among adolescents is declining year by year and has become an important public health issue of concern to the world (16–18). Research has shown that about 80% of adolescents around the world have insufficient levels of daily physical activity, far below the World Health Organization’s recommendation of at least 1 h of moderate to vigorous physical activity per day. At the same time, 25% of adolescents spend more than 3 h a day in sedentary positions, in addition to being at school and completing their homework (19). It has also been found that the problem of physical inactivity among Chinese youth is particularly pronounced, with only 15–34% of students meeting the physical activity guideline recommendations and less than a quarter (22%) of schoolchildren engaging in physical activity for 60 min or more per day; in addition (20). However, past research has focused primarily on physical activity levels in adolescents, and relatively limited research has been conducted on the association between exercise duration and psychological symptoms. Studies have also shown that intake of UPF may increase the risk of psychological symptoms by triggering an inflammatory response, while exercise can offset the negative impact of UPF on psychological symptoms by enhancing neuroplasticity and anti-inflammatory effects (21).

Given the associations between dietary behaviors and physical activity, and psychological symptoms. Past research seems to have little literature explored the combined effects of diet and physical activity on psychological symptoms. However, the association between UPF consumption and the duration of exercise with psychological symptoms is unclear. In particular, no evidence has been found to investigate the association between UPF consumption and duration of exercise with psychological symptoms among Chinese adolescents nationwide. In this study, a cross-sectional assessment of UPF consumption, duration of exercise, and psychological symptoms was conducted among 14,445 adolescents aged 13–18 years in five geographic regions of China. The goal was to examine how the intake of UPF and exercise duration are linked to psychological symptoms in Chinese teenagers. This research offers valuable insights and a solid foundation for preventing and addressing psychological issues among Chinese adolescents.

## 2 Methods

### 2.1 Study participants and procedure

Participants were selected for this study in 2023 using stratified whole cluster random sampling. The participant selection process was divided into three stages. In the first stage, considering the geographical distribution of cities in China, this study selected participants in five cities in China: Changchun City, Jilin Province, in the northern region; Fuzhou City, Fujian Province, in the southern region; Shangrao City, Jiangxi Province, in the central region; Urumqi City, Xinjiang Province, in the western region; and Suzhou City, Jiangsu Province, in the eastern region, as the participant-selection areas. In the second stage, two middle schools were selected in each city and village, and four middle schools were selected in each city as the test schools for this study. In the third stage, in each secondary school, three teaching classes were randomly selected according to the sampling unit of class in each grade from the first grade to the third grade of senior high school, i.e., a total of 18 teaching classes were randomly selected from each school.

**Figure 1:**
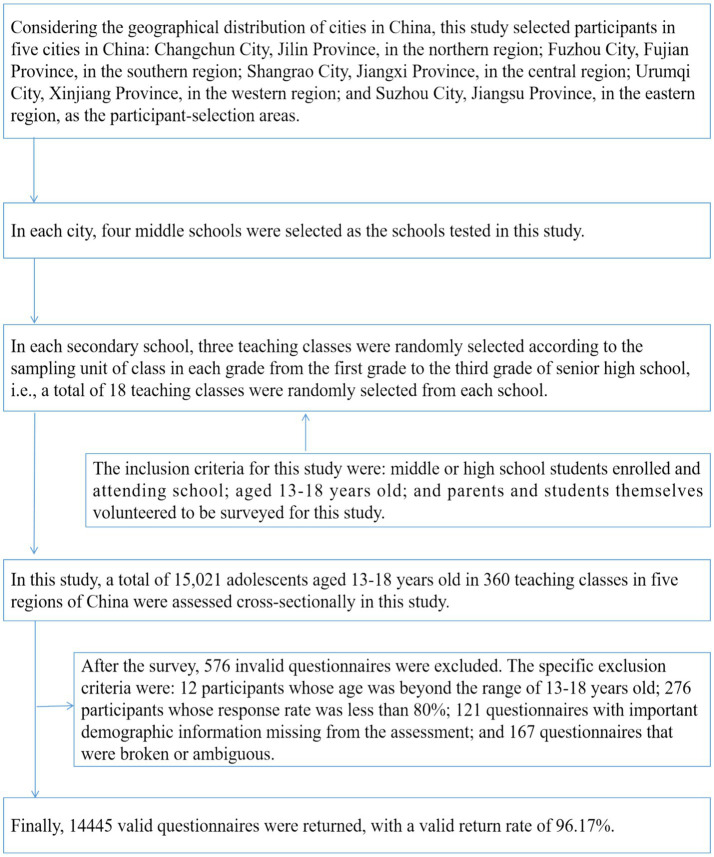
Participant extraction process for Chinese adolescents.

The inclusion criteria for this study were: middle or high school students enrolled and attending school; aged 13–18 years old; and parents and students themselves volunteered to be surveyed for this study. A total of 15,021 adolescents aged 13–18 years old in 360 teaching classes in five regions of China were assessed cross-sectionally in this study. After the survey, 576 invalid questionnaires were excluded. The specific exclusion criteria were: 12 participants whose age was beyond the range of 13–18 years old; 276 participants whose response rate was less than 80%; 121 questionnaires with important demographic information missing from the assessment; and 167 questionnaires that were broken or ambiguous. Finally, 14,445 valid questionnaires were returned, with a valid return rate of 96.17%. Figure 1 shows the participant extraction process of this study.

This study was conducted by the Declaration of Helsinki and approved by the Human Ethics Committee of Jiangxi Science and Technology Normal University (IRB-JXSTNU-2022003).

### 2.2 Ultra-processed food (UPF) consumption

In this study, UPF consumption was assessed using the 136-item semi-quantitative food-frequency questionnaire (FFQ), which has been repeatedly validated in several studies (22–24). FFQ was developed based on the methodology proposed by Willett (25). The FFQ details the frequency and portion size of each food consumed. The FFQ consists of 72 foods that were commonly consumed during the previous month and includes the following three sections: food list, frequency response, and average intake (25). Combined with past research classifications, frequency responses in this study included the following four categories: <3 Times/week, 3–4 times/week, 5–6 times/week, and >6 times/week (26). There are no standardized food portion sizes in China, so after surveying local food types and portion sizes in the marketplace, the use of visual aids, including photographs of utensils and food portions, was used, with each serving calculated as 50 g. Weekly UPF consumption was estimated by frequency.

The UPF in this study was determined based on NOVA classification definitions (11). The classification standard is based on the degree and purpose of industrial processing and divides foods into four categories. The first category is unprocessed or minimally processed foods, including fruits, vegetables, grains, nuts, natural yogurt without added sugar, and fresh and pasteurized milk. The second category is processed cooking ingredients such as salt, soy sauce, vinegar, sugar, honey, lard, vegetable oil, and consumable oil. The third category is processed foods, such as filled vegetables, fruits, and beans, filled fish, cheese, bread, and salted or sweetened nuts of all types. The fourth category is UPF, including all types of sugary drinks, carbonated drinks, functional drinks, fruit drinks, processed meats, cookies, candies, chocolates, instant packaged noodles, and soups, all types of sweet or salty snacks, potato chips, and sweetened milk (11). Take Coca-Cola, for example. It’s packed with artificial flavors, colors, and tons of refined sugar. It goes through a bunch of industrial processes and barely has any whole food ingredients.

These foods are classified as UPF because they undergo multiple stages of industrial processing, including refining, mixing, and adding artificial ingredients. They contain high amounts of artificial additives such as preservatives, artificial flavors, and colors. Designed for convenience, they often have a long shelf life and are ready-to-eat or easy to prepare. Additionally, they typically have a high sugar, salt, or fat content to enhance taste and texture, and they lack whole food content, being far removed from their natural state and often containing refined ingredients. See Supplementary Table 1 for specific classification methods.

### 2.3 Duration of exercise

The assessment of the duration of exercise in this study was calculated using the China National Survey on Students’ Constitution and Health (CNSSCH) on the duration of physical activity (27, 28). Primarily assesses the average time per day that participants have engaged in moderate-to-vigorous physical activity (MVPA) over the past 7 days. Includes a variety of ball games (basketball, soccer, volleyball, tennis, badminton, table tennis), calisthenics, running, brisk walking, swimming, skiing, skating, outdoor hiking, biking, and other exercise programs. Include the average length of exercise per day and the frequency of exercise per week. It was filled in according to weekdays, Monday through Friday, and rest days, Saturday and Sunday, and the average daily length of participants’ MVPA participation in the past 7 days was calculated. In this study, duration of exercise was categorized into four categories based on the actuality of the study: <30 min/day, 30–60 min/day, 61–120 min/day, and >120 min/day (29).

### 2.4 Psychological symptoms

In this study, adolescent psychological symptoms were assessed using the Brief Instrument on Psychological Health of Youths (BIOPHY) questionnaire. This questionnaire has good reliability and validity for the assessment of psychological symptoms in Chinese adolescents and has been used in several studies (30). The Cronbach’s a coefficient of the questionnaire was 0.93. The questionnaire consisted of 3 dimensions, namely, emotional problems, behavioral problems, and social adjustment difficulties, and consisted of 15 items, with 6 options for each item, ranging from 1 to 6 points, from “lasting more than 3 months” to “none or lasting less than a week,” respectively. The higher the score, the shorter the duration of the psychological symptoms, and the longer the duration of the symptoms ≥ 1 month, the shorter the duration of the psychological symptoms, and the longer the duration of the symptoms ≥ 1 month, the longer the duration of the psychological symptoms (30). Psychological symptoms were defined as the presence of psychological subhealth, i.e., the presence of psychological symptoms, and P_90_ was used as a criterion for defining adolescents’ psychological symptom problems in the three dimensions, with <P_90_ being healthy and ≥P_90_ being unhealthy (30).

### 2.5 Covariates

The assessment of covariates in this study included physical measures and indicators of participants’ lifestyles. Physical measures included height, weight, grip strength, and standing long jump. Test methods and instruments were based on the methods and instruments specified in the China National Survey on Students’ Constitution and Health (CNSSCH). The BMI of the participants was calculated based on height and weight. BMI was calculated by dividing weight (kilograms) by the square of height (meters) (kg/m^2^). The BMI of the participants was categorized as Slimmer, Normal, Overweight, and Obese. Adolescent BMI was categorized using the Working Group on Obesity in China (WGOC) criteria, with cut-off points at the 85th and 95th percentiles of BMI (Overweight: 85th percentile < BMI < 95th percentile; Obese: 95th percentile < BMI) (31). Definition of adolescent “slimmer” based on age-sex BMI cutoffs for malnutrition in Chinese adolescents (32).

Participants’ lifestyles were assessed in terms of the father’s education and the mother’s education, which were categorized into elementary school and below, middle school, high school, and college and above. Commuting style includes two categories: positive style and negative style. The positive style includes walking and bicycling to and from school. Negative style includes commuting to and from school by bus, bicycle, or subway. The sleep duration was calculated based on the participants’ sleep time and wake-up time (33). Based on the classification of several studies, they were categorized as <8 h/d, ≥8 h/d (34).

### 2.6 Statistical analysis

For this study of participant characteristics, continuous variables were expressed as means and standard deviations, and count data were expressed as percentages. The chi-square test was used to compare the prevalence of psychological symptoms among adolescents with different demographic characteristics, UPF consumption, and duration of exercise. Because the dependent variables in this study are classified into two categories, binary logistic regression analysis was used to analyze the association of UPF consumption and duration of exercise with the prevalence of psychological symptoms and dimensions (emotional problems, behavioral problems, social adjustment difficulties). Based on previous data and combined with this study, some factors that affect participants’ psychological symptoms were adjusted as covariates and analyzed. Model 1 was not adjusted for covariates, while Model 2 was adjusted for age, father’s education, and mother’s education; Model 3 was adjusted for commuting style, sleep duration, BMI, and grip strength based on Model 2, Model 3 adjusted commuting style, sleep duration, BMI, grip strength, and standing long jump based on Model 2. To further understand the association between the joint effect of UPF consumption and duration of exercise with psychological symptoms in Chinese adolescents, the present study was conducted using ordered logistic regression analysis in a generalized linear model. The Model adjusted for age, father’s education, mother’s education, commuting style, sleep duration, BMI, grip strength, and standing long jump. The odds ratio (OR) and 95% confidence interval (CI) were reported after logistic regression analysis. Statistical analysis was performed using SPSS 25.0 software. *p* < 0.05 was used as the test level.

## 3 Results

In this study, 14,445 (7,240 boys, 50.12%) Chinese adolescents aged 13–18 years were surveyed and analyzed cross-sectionally. The mean age of the participants in this study was (15.50 ± 1.71) years. The results of this study showed that overall, the prevalence of emotional problems, behavioral problems, and social adjustment difficulties among Chinese adolescents was 28.2, 28.0, and 17.6%, respectively; the prevalence of psychological symptoms among Chinese adolescents was 22.0%. In terms of sex, the prevalence rates of behavioral problems, social adjustment difficulties, and psychological symptoms were higher for boys than for girls, and the differences were statistically significant (*χ*^2^ values of 6.756, 16.306, 6.044, *p* < 0.05). The proportion of UPF consumption frequency ≥5 times/week in this study was Coca-Cola, Pepsi, Instant Ramen, Lays Classic, Potato Chips, Granola Bars, Deli Ham, Snickers, Oreos, Mini-Wheats, and Ice Cream, respectively, with the proportions being 7.9, 6.5, 6.1, 5.9, 5.2, 4.8, 4.5, 4.1, 3.9, and 3.5%, respectively. Table 1 also shows the basic characteristics of Chinese participants of different sexes.

**Table 1:** Basic characteristics of Chinese adolescent participants.

Characteristic	Boys	Girls	*χ*^2^/*t*-value	*P*-value	Total
Numbers	7,240	7,205			14,445
Age (M ± SD)	15.50 ± 1.71	15.50 ± 1.71	0.134	0.893	15.50 ± 1.71
Height (M ± SD)	171.03 ± 8.16	161.58 ± 6.36	77.558	<0.001	166.32 ± 8.71
Weight (M ± SD)	60.21 ± 12.85	51.64 ± 8.30	47.553	<0.001	55.94 ± 11.64
BMI (M ± SD)	20.47 ± 3.58	19.75 ± 2.81	13.423	<0.001	20.11 ± 3.24
Grip strength (M ± SD)	37.03 ± 9.73	25.79 ± 6.05	83.317	<0.001	31.43 ± 9.86
Standing long jump (M ± SD)	209.67 ± 29.84	167.08 ± 20.91	99.282	<0.001	188.42 ± 33.43
Father’s education [*N* (%)]			8.916	0.03	
Elementary school and below	912(12.6)	952(13.2)			1864(12.9)
Middle School	2,598(35.9)	2,565(35.6)			5,163(35.7)
High School	2,388(33.0)	2,474(34.3)			4,862(33.7)
College and above	1,342(18.5)	1,214(16.8)			2,556(17.7)
Mother’s education [*N* (%)]			21.230	<0.001	
Elementary school and below	1,298(17.9)	1,328(18.4)			2,626(18.2)
Middle School	2,442(33.7)	2,583(35.9)			5,025(34.8)
High School	2,248(31.0)	2,240(31.1)			4,488(31.1)
College and above	1,252(17.3)	1,054(14.6)			2,306(16)
Commuting style [*N* (%)]			32.033	<0.001	
Positive style	3,906(54.0)	3,548(49.2)			7,454(51.6)
Negative style	3,334(46.0)	3,657(50.8)			6,991(48.4)
Sleep duration [*N* (%)]			58.199	<0.001	
<8 h/day	6,382(88.1)	6,625(92.0)			13,007(90)
≥8 h/day	858(11.9)	580(8.0)			1,438(10)
BMI Classification [*N* (%)]			452.490	<0.001	
Slimmer	1,064(14.7)	704(9.8)			1768(12.2)
Normal	4,772(65.9)	5,819(80.8)			10,591(73.3)
Overweight	970(13.4)	548(7.6)			1,518(10.5)
Obese	434(6.0)	134(1.9)			568(3.9)
Grip strength quartiles [*N* (%)]			4915.418	<0.001	
Q1	768(10.6)	2,944(40.9)			3,712(25.7)
Q2	928(12.8)	2,602(36.1)			3,530(24.4)
Q3	2,198(30.4)	1,413(19.6)			3,611(25.0)
Q4	3,346(46.2)	246(3.4)			3,592(24.9)
Standing long jump quartiles [*N* (%)]			6187.177	<0.001	
Q1	520(7.2)	3,119(43.3)			3,639(25.2)
Q2	922(12.7)	2,728(37.9)			3,650(25.3)
Q3	2,340(32.3)	1,230(17.1)			3,570(24.7)
Q4	3,458(47.8)	128(1.8)			3,586(24.8)
Ultra-processed food [*N* (%)]			84.065	<0.001	
<3 times/week	3,602(49.8)	3,815(52.9)			7,417(51.3)
3–4 times/week	2,232(30.8)	2,370(32.9)			4,602(31.9)
5–6 times/week	924(12.8)	740(10.3)			1,664(11.5)
>6 times/week	482(6.7)	280(3.9)			762(5.3)
Duration of exercise [*N* (%)]			802.046	<0.001	
<30 min/day	2,642(36.5)	4,093(56.8)			6,735(46.6)
30–60 min/day	3,129(43.2)	2,569(35.7)			5,698(39.4)
61–120 min/day	1,094(15.1)	442(6.1)			1,536(10.6)
>120 min/day	375(5.2)	101(1.4)			476(3.3)
Emotional problems [*N* (%)]	2055(28.4)	2017(28.0)	0.271	0.603	4,072(28.2)
Behavioral problems [*N* (%)]	2095(28.9)	1945(27.0)	6.756	0.009	4,040(28.0)
Social adjustment difficulties [*N* (%)]	1,367(18.9)	1,176(16.3)	16.306	<0.001	2,543(17.6)
Psychological symptoms [*N* (%)]	1,651(22.8)	1,521(21.1)	6.044	0.014	3,172(22.0)

Table 2 shows the characterization of the prevalence of psychological symptoms and the prevalence of the three dimensions (emotional problems, behavioral problems, and social adjustment difficulties) among Chinese adolescents. The results of this study showed that across sex, father’s education, commuting style, sleep duration, BMI classification, ultra-processed food, and duration of exercise, the prevalence of There were significant differences in the prevalence of psychological symptoms (*χ*^2^ values of 6.044, 35.203, 11.099, 19.496, 13.133, 53.166, 111.716, respectively, *p* < 0.05). The results in Table 2 also showed that girls (21.1%), father’s education as high school (20.2%), commuting style as positive style (20.8%), sleep duration as ≥8 h/d (17.4%), BMI classification of overweight (20.7%), ultra-processed food of 3–4 times/week (20.0%), and duration of exercise of 30–60 min/day (18.4%), the prevalence of psychological symptoms was the lowest among Chinese adolescents.

**Table 2:** Characterization of adolescents’ psychological symptoms in China [*N* (%)].

Characteristic	Emotional problems	*χ*^2^-value	*P*-value	Behavioral problems	*χ*^2^-value	*P*-value	Social adjustment difficulties	*χ*^2^-value	*P*-value	Psychological symptoms	*χ*^2^-value	*P*-value
Sex		0.271	0.603		6.756	0.009		16.306	<0.001		6.044	0.014
Boys	2055(28.4)			2095(28.9)			1,367(18.9)			1,651(22.8)		
Girls	2017(28.0)			1945(27.0)			1,176(16.3)			1,521(21.1)		
Father’s education		28.474	<0.001		42.550	<0.001		35.872	<0.001		35.203	<0.001
Elementary school and below	605(32.5)			631(33.9)			415(22.3)			501(26.9)		
Middle School	1,389(26.9)			1,395(27.0)			896(17.4)			1,117(21.6)		
High School	1,312(27.0)			1,276(26.2)			782(16.1)			984(20.2)		
College and above	766(30.0)			738(28.9)			450(17.6)			570(22.3)		
Mother’s education		1.902	0.593		10.917	0.012		10.727	0.013		6.275	0.099
Elementary school and below	731(27.8)			787(30.0)			479(18.2)			595(22.7)		
Middle School	1,449(28.8)			1,425(28.4)			922(18.3)			1,143(22.7)		
High School	1,240(27.6)			1,186(26.4)			788(17.6)			962(21.4)		
College and above	652(28.3)			642(27.8)			354(15.4)			472(20.5)		
Commuting style		9.042	0.003		3.543	0.060		8.389	0.004		11.099	0.001
Positive style	2020(27.1)			2034(27.3)			1,246(16.7)			1,554(20.8)		
Negative style	2052(29.4)			2006(28.7)			1,297(18.6)			1,618(23.1)		
Sleep duration		27.803	<0.001		16.791	<0.001		26.957	<0.001		19.496	<0.001
<8 h/day	3,752(28.8)			3,704(28.5)			2,361(18.2)			2,922(22.5)		
≥8 h/day	320(22.3)			336(23.4)			182(12.7)			250(17.4)		
BMI Classification		8.175	0.043		14.329	0.002		15.790	0.001		13.133	0.004
Slimmer	514(29.1)			526(29.8)			322(18.2)			414(23.4)		
Normal	2,980(28.1)			2,948(27.8)			1831(17.3)			2,290(21.6)		
Overweight	396(26.1)			382(25.2)			256(16.9)			314(20.7)		
Obese	182(32.0)			184(32.4)			134(23.6)			154(27.1)		
Grip strength quartiles		2.061	0.560		4.641	0.200		6.250	0.100		0.171	0.982
Q1	1,066(28.7)			1,058(28.5)			642(17.3)			822(22.1)		
Q2	990(28.0)			1,022(29.0)			582(16.5)			768(21.8)		
Q3	1,033(28.6)			971(26.9)			650(18.0)			795(22.0)		
Q4	983(27.4)			989(27.5)			669(18.6)			787(21.9)		
Standing long jump quartiles		10.183	0.017		3.084	0.379		9.748	0.021		1.435	0.697
Q1	1,077(29.6)			1,035(28.4)			638(17.5)			811(22.3)		
Q2	1,060(29.0)			1,036(28.4)			604(16.5)			788(21.6)		
Q3	950(26.6)			958(26.8)			612(17.1)			768(21.5)		
Q4	985(27.5)			1,011(28.2)			689(19.2)			805(22.4)		
Ultra-processed food		52.570	<0.001		94.246	<0.001		65.492	<0.001		53.166	<0.001
<3 times/week	2,109(28.4)			2054(27.7)			1,311(17.7)			1,617(21.8)		
3–4 times/week	1,182(25.7)			1,157(25.1)			707(15.4)			922(20.0)		
5–6 times/week	491(29.5)			513(30.8)			319(19.2)			393(23.6)		
>6 times/week	290(38.1)			316(41.5)			206(27.0)			240(31.5)		
Duration of exercise		141.696	<0.001		45.660	<0.001		114.601	<0.001		111.716	<0.001
<30 min/day	2,219(32.9)			2059(30.6)			1,428(21.2)			1741(25.9)		
30–60 min/day	1,356(23.8)			1,441(25.3)			827(14.5)			1,050(18.4)		
61–120 min/day	380(24.7)			402(26.2)			230(15.0)			292(19.0)		
>120 min/day	117(24.6)			138(29.0)			58(12.2)			89(18.7)		

Table 3 shows the univariate analysis of UPF consumption, duration of exercise, and psychological symptoms in Chinese adolescents. Overall, the results showed that the prevalence of psychological symptoms among Chinese adolescents with UPF consumption of <3 times/week, 3–4 times/week, 5–6 times/week, and >6 times/week were 21.8, 20.0, 23.6, and 31.5%, respectively, showing an overall trend of increasing prevalence, and the difference was statistically significant (*χ*^2^ value of 53.166, *p* < 0.001). The same trend was observed in the dimensions of emotional problems, behavioral problems, and social adjustment difficulties.

**Table 3:** One-way analysis of UPF consumption, duration of exercise with psychological symptoms in Chinese adolescents.

Variable	*N*	Emotional problems			Behavioral problems			Social adjustment difficulties			Psychological symptoms		
		*N* (%)	*χ*^2^-value	*P-*value	*N* (%)	*χ*^2^-value	*P*-value	*N* (%)	*χ*^2^-value	*P*-value	*N* (%)	*χ*^2^-value	*P*-value
Boys													
Ultra-processed food			29.188	<0.001		75.019	<0.001		37.127	<0.001		27.258	<0.001
<3 times/week	3,602	1,058(29.4)			996(27.7)			672(18.7)			816(22.7)		
3–4 times/week	2,232	568(25.4)			596(26.7)			374(16.8)			468(21.0)		
5–6 times/week	924	251(27.2)			283(30.6)			183(19.8)			213(23.1)		
>6 times/week	482	178(36.9)			220(45.6)			138(28.6)			154(32.0)		
Duration of exercise			88.700	<0.001		26.313	<0.001		65.914	<0.001		58.265	<0.001
<30 min/day	2,642	922(34.9)			850(32.2)			626(23.7)			732(27.7)		
30–60 min/day	3,129	785(25.1)			846(27.0)			524(16.7)			639(20.4)		
61–120 min/day	1,094	252(23.0)			280(25.6)			166(15.2)			204(18.6)		
>120 min/day	375	96(25.6)			119(31.7)			51(13.6)			76(20.3)		
Girls													
Ultra-processed food			32.749	<0.001		28.162	<0.001		24.756	<0.001		25.562	<0.001
<3 times/week	3,815	1,051(27.5)			1,058(27.7)			639(16.7)			801(21.0)		
3–4 times/week	2,370	614(25.9)			561(23.7)			333(14.1)			454(19.2)		
5–6 times/week	740	240(32.4)			230(31.1)			136(18.4)			180(24.3)		
>6 times/week	280	112(40.0)			96(34.3)			68(24.3)			86(30.7)		
Duration of exercise			72.907	<0.001		36.111	<0.001		78.270	<0.001		75.634	<0.001
<30 min/day	4,093	1,297(31.7)			1,209(29.5)			802(19.6)			1,009(24.7)		
30–60 min/day	2,569	571(22.2)			595(23.2)			303(11.8)			411(16.0)		
61–120 min/day	442	128(29.0)			122(27.6)			64(14.5)			88(19.9)		
>120 min/day	101	21(20.8)			19(18.8)			7(6.9)			13(12.9)		
Total													
Ultra-processed food			52.570	<0.001		94.246	<0.001		65.492	<0.001		53.166	<0.001
<3 times/week	7,417	2,109(28.4)			2054(27.7)			1,311(17.7)			1,617(21.8)		
3–4 times/week	4,602	1,182(25.7)			1,157(25.1)			707(15.4)			922(20.0)		
5–6 times/week	1,664	491(29.5)			513(30.8)			319(19.2)			393(23.6)		
>6 times/week	762	290(38.1)			316(41.5)			206(27.0)			240(31.5)		
Duration of exercise			141.696	<0.001		45.66	<0.001		114.601	<0.001		111.716	<0.001
<30 min/day	6,735	2,219(32.9)			2059(30.6)			1,428(21.2)			1741(25.9)		
30–60 min/day	5,698	1,356(23.8)			1,441(25.3)			827(14.5)			1,050(18.4)		
61–120 min/day	1,536	380(24.7)			402(26.2)			230(15.0)			292(19.0)		
>120 min/day	476	117(24.6)			138(29.0)			58(12.2)			89(18.7)		

The results in Table 3 also showed that the prevalence of psychological symptoms among Chinese adolescents with different durations of exercise (<30 min/day, 30–60 min/day, 61–120 min/day, >120 min/day) were 25.9, 18.4, 19.0, and 18.7%, respectively. Overall, the prevalence of psychological symptoms showed a decreasing trend with the prolongation of the duration of exercise in adolescents, and the difference was statistically significant in comparison (*χ*^2^ value of 111.716, *p* < 0.001). The same trend was observed for emotional problems, behavioral problems, social adjustment difficulties dimensions, and prevalence of psychological symptoms across sexes (*p* < 0.001).

Table 4 shows the results of binary logistic regression analysis of UPF consumption and duration of exercise with psychological symptoms in Chinese adolescents. In this study, the prevalence of psychological symptoms among Chinese adolescents was used as the dependent variable. Binary logistic regression analyses were conducted with UPF consumption and duration of exercise as independent variables. Model 1 was not adjusted for covariates, Model 2 was adjusted for age, father’s education, and mother’s education; Model 3 was adjusted for commuting style, sleep duration, BMI, and grip strength based on Model 2, and standing long jump. The results of Model 3 showed that, overall, the prevalence of psychological symptoms was significantly higher in adolescents with UPF consumption >6 times/week (OR = 1.72, 95% CI: 1.46–2.03), using the UPF consumption <3 times/week group as a reference (*p* < 0.001). In terms of duration of exercise, with duration of exercise >120 min/day as the reference group, the prevalence of psychological symptoms was also significantly higher in adolescents with a duration of exercise <30 min/day (OR = 1.53, 95% CI: 1.21 ~ 1.95) (*p* < 0.001). The same trend existed when analyzed stratified by sex (*p* < 0.05).

**Table 4:** Binary logistic regression analysis of UPF consumption, duration of exercise with psychological symptoms in Chinese adolescents.

Sex/variable		Model 1		Model 2		Model 3	
		OR (95% CI)	*P -*value	OR (95% CI)	*P -*value	OR (95% CI)	*P -*value
Boys							
Ultra-processed food	<3 times/week	1.00		1.00		1.00	
	3–4 times/week	0.91(0.80 ~ 1.03)	0.125	0.90(0.79 ~ 1.02)	0.102	0.90(0.79 ~ 1.03)	0.124
	5–6 times/week	1.02(0.86 ~ 1.21)	0.810	1.01(0.85 ~ 1.20)	0.896	1.03(0.87 ~ 1.23)	0.702
	>6 times/week	1.60(1.30 ~ 1.97)	<0.001	1.58(1.28 ~ 1.94)	<0.001	1.65(1.33 ~ 2.03)	<0.001
Duration of exercise	>120 min/day	1.00		1.00		1.00	
	61–120 min/day	0.90(0.67 ~ 1.21)	0.491	0.89(0.66 ~ 1.20)	0.444	0.88(0.66 ~ 1.19)	0.408
	30–60 min/day	1.01(0.77 ~ 1.32)	0.939	0.99(0.76 ~ 1.29)	0.933	0.96(0.73 ~ 1.25)	0.738
	<30 min/day	1.51(1.16 ~ 1.97)	0.002	1.48(1.14 ~ 1.94)	0.004	1.43(1.10 ~ 1.88)	0.009
Girls							
Ultra-processed food	<3 times/week	1.00		1.00		1.00	
	3–4 times/week	0.89(0.78 ~ 1.01)	0.080	0.89(0.78 ~ 1.01)	0.076	0.89(0.79 ~ 1.02)	0.089
	5–6 times/week	1.21(1.01 ~ 1.46)	0.044	1.22(1.01 ~ 1.47)	0.035	1.23(1.02 ~ 1.48)	0.029
	>6 times/week	1.67(1.28 ~ 2.18)	<0.001	1.68(1.29 ~ 2.20)	<0.001	1.72(1.31 ~ 2.25)	<0.001
Duration of exercise	>120 min/day	1.00		1.00		1.00	
	61–120 min/day	1.68(0.90 ~ 3.15)	0.104	1.61(0.86 ~ 3.02)	0.137	1.62(0.86 ~ 3.05)	0.134
	30–60 min/day	1.29(0.71 ~ 2.33)	0.400	1.29(0.71 ~ 2.33)	0.407	1.26(0.69 ~ 2.28)	0.453
	<30 min/day	2.22(1.23 ~ 3.98)	0.008	2.27(1.26 ~ 4.08)	0.006	2.20(1.22 ~ 3.97)	0.009
Total							
Ultra-processed food	<3 times/week	1.00		1.00		1.00	
	3–4 times/week	0.90(0.82 ~ 0.98)	0.020	0.9(0.82 ~ 0.98)	0.018	0.91(0.83 ~ 1.00)	0.039
	5–6 times/week	1.11(0.98 ~ 1.26)	0.109	1.10(0.97 ~ 1.25)	0.133	1.13(1.00 ~ 1.28)	0.056
	>6 times/week	1.65(1.40 ~ 1.94)	<0.001	1.64(1.39 ~ 1.92)	<0.001	1.72(1.46 ~ 2.03)	<0.001
Duration of exercise	>120 min/day	1.00		1.00		1.00	
	61–120 min/day	1.02(0.78 ~ 1.33)	0.879	1.01(0.78 ~ 1.32)	0.916	1.02(0.78 ~ 1.33)	0.880
	30–60 min/day	0.98(0.77 ~ 1.25)	0.887	0.98(0.77 ~ 1.25)	0.868	0.98(0.77 ~ 1.25)	0.852
	<30 min/day	1.52(1.20 ~ 1.92)	0.001	1.53(1.21 ~ 1.94)	<0.001	1.53(1.21 ~ 1.95)	<0.001

Model 1 did not adjust for covariates, Model 2 adjusted for age, father’s education, and mother’s education; based on Model 2, Model 3 adjusted for commuting style, sleep duration, BMI, grip strength, and standing long jump.

**Figure 2:**
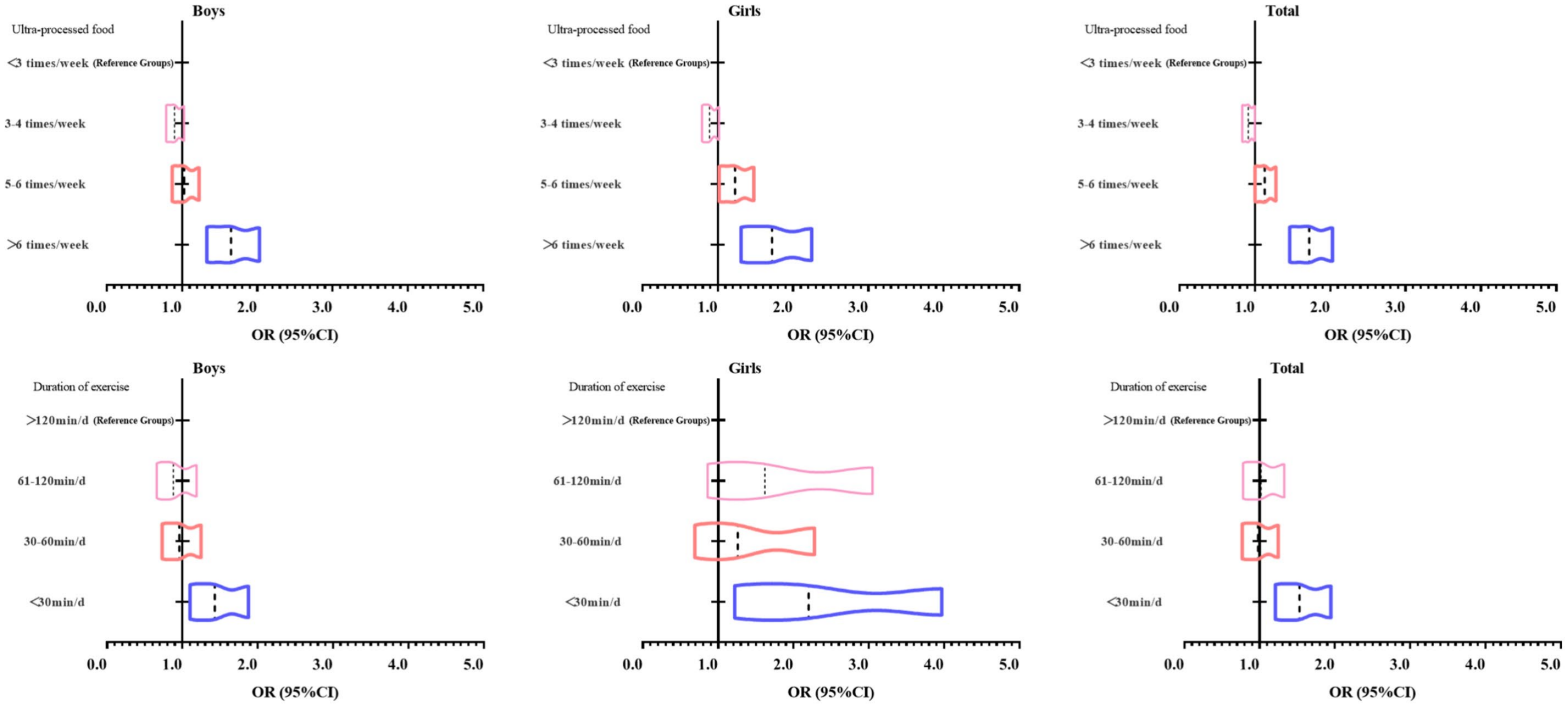
Binary logistic regression analysis of the OR value change trend of UPF consumption, duration of exercise with psychological symptoms in Chinese adolescents.

As shown in Figure 2, as the UPF consumption continues to increase, the risk of psychological symbols in adolescents shows an increase, that is, the OR value gradually increases. Similarly, as the duration of adolescents continues to decrease, the risk of psychological symptoms in adolescents also shows a continuous increase, and the OR value gradually increases, that is, it shifts more to the right. Boys and girls have the same trend.

**Figure 3:**
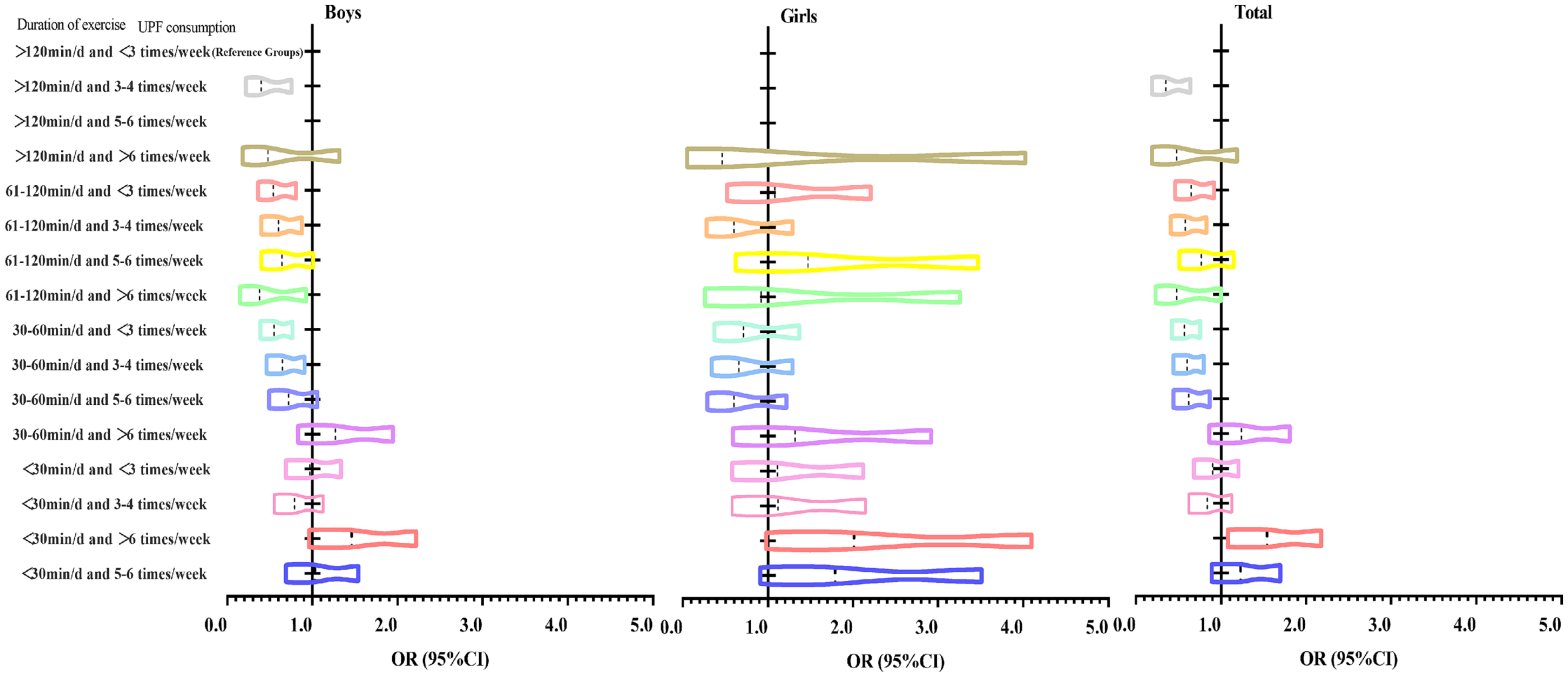
Trends in ORs of ordered logistic regression analysis of UPF consumption, duration of exercise with psychological symptoms in Chinese adolescents.

Table 5 shows the ordered logistic regression analyses of UPF consumption and duration of exercise with psychological symptoms in Chinese adolescents. The model adjusted for age, father’s education, mother’s education, commuting style, sleep duration, BMI, grip strength, and standing long jump. Overall, the results showed that using adolescents in the duration of exercise >120 min/day and UPF consumption <3 times/week group as the reference group ordered logistic regression analysis revealed that the prevalence of psychological symptoms was significantly higher in the adolescents with the duration of exercise <30 min/day and UPF consumption >6 times/week group (OR = 1.54, 95% CI: 1.08 ~ 2.18) (*p* < 0.05). However, the same trend existed in the analysis stratified by gender, but the results were not significant. The change trend of OR value is shown in Figure 3.

**Table 5:** Ordered logistic regression analysis of UPF consumption, duration of exercise with psychological symptoms in Chinese adolescents.

Sex	Variable		Ordered logistic regression	
	Duration of exercise	Ultra-processed food	OR (95% CI)	*P-*value
Boys	>120 min/day	<3 times/week	1.00	
		3–4 times/week	0.40(0.22 ~ 0.76)	0.005
		5–6 times/week	–	–
		>6 times/week	0.48(0.18 ~ 1.32)	0.154
	61–120 min/day	<3 times/week	0.54(0.36 ~ 0.81)	0.003
		3–4 times/week	0.60(0.40 ~ 0.88)	0.010
		5–6 times/week	0.64(0.40 ~ 1.01)	0.057
		>6 times/week	0.38(0.15 ~ 0.93)	0.035
	30–60 min/day	<3 times/week	0.55(0.39 ~ 0.77)	0.001
		3–4 times/week	0.65(0.46 ~ 0.91)	0.013
		5–6 times/week	0.72(0.49 ~ 1.06)	0.097
		>6 times/week	1.27(0.83 ~ 1.95)	0.274
	<30 min/day	<3 times/week	0.97(0.69 ~ 1.34)	0.831
		3–4 times/week	0.79(0.55 ~ 1.13)	0.188
		5–6 times/week	1.03(0.69 ~ 1.54)	0.887
		>6 times/week	1.46(0.96 ~ 2.22)	0.076
Girls	>120 min/day	<3 times/week	1.00	
		3–4 times/week	–	–
		5–6 times/week	–	–
		>6 times/week	0.46(0.05 ~ 4.03)	0.482
	61–120 min/day	<3 times/week	1.08(0.52 ~ 2.21)	0.843
		3–4 times/week	0.60(0.28 ~ 1.29)	0.186
		5–6 times/week	1.47(0.62 ~ 3.47)	0.384
		>6 times/week	0.92(0.26 ~ 3.26)	0.893
	30–60 min/day	<3 times/week	0.71(0.37 ~ 1.37)	0.312
		3–4 times/week	0.66(0.34 ~ 1.29)	0.226
		5–6 times/week	0.60(0.29 ~ 1.22)	0.158
		>6 times/week	1.32(0.59 ~ 2.92)	0.497
	<30 min/day	<3 times/week	1.11(0.58 ~ 2.12)	0.749
		3–4 times/week	1.12(0.58 ~ 2.15)	0.732
		5–6 times/week	1.79(0.91 ~ 3.51)	0.091
		>6 times/week	2.01(0.98 ~ 4.10)	0.056
Total	>120 min/day	<3 times/week	1.00	
		3–4 times/week	0.35(0.19 ~ 0.64)	0.001
		5–6 times/week	–	–
		>6 times/week	0.48(0.19 ~ 1.19)	0.112
	61–120 min/day	<3 times/week	0.65(0.46 ~ 0.92)	0.014
		3–4 times/week	0.58(0.41 ~ 0.83)	0.002
		5–6 times/week	0.77(0.51 ~ 1.15)	0.202
		>6 times/week	0.48(0.23 ~ 1.00)	0.050
	30–60 min/day	<3 times/week	0.57(0.42 ~ 0.76)	<0.001
		3–4 times/week	0.60(0.44 ~ 0.80)	0.001
		5–6 times/week	0.62(0.44 ~ 0.87)	0.006
		>6 times/week	1.24(0.86 ~ 1.81)	0.255
	<30 min/day	<3 times/week	0.90(0.68 ~ 1.21)	0.489
		3–4 times/week	0.84(0.62 ~ 1.13)	0.238
		5–6 times/week	1.23(0.89 ~ 1.70)	0.213
		>6 times/week	1.54(1.08 ~ 2.18)	0.016

The Model adjusted age, father’s education, mother’s education, commuting style, sleep duration, BMI, grip strength, standing long jump.

## 4 Discussion

In this study, we analyzed for the first time the association between UPF consumption, and duration of exercise with psychological symptoms in Chinese adolescents using a national sample of adolescents. We found that the prevalence of psychological symptoms was higher among Chinese adolescents with higher UPF consumption. This finding is consistent with the findings of several previous studies (10, 35). In addition, the present study showed that there was an association between the duration of exercise and the prevalence of psychological symptoms. The longer the duration of exercise, the lower the prevalence of psychological symptoms in adolescents. However, this study did not find any significant difference between the combined effect of UPF consumption and duration of exercise on psychological symptoms when stratified by sex. The results of this study are different from those of past studies.

Studies have shown that increased UPF consumption in adolescents is an important risk factor for the development of overweight or obesity and that the development of overweight and obesity is also an important cause of various psychiatric disorders (36). There are also studies confirming that the majority of UPF consumption among adolescents is centered on the consumption of sugar-sweetened beverages and that excessive consumption of sugar-sweetened beverages is associated with the development of several psychiatric disorders (37, 38). A survey of Chinese adolescents shows that excessive intake of sugary drinks leads to obesity, changes in the microbial environment of the intestinal flora, and affects the secretion of endorphin hormones in the body, thus causing the occurrence of mental illness (39). The results of the present study also confirm that the higher prevalence of psychological symptoms in adolescents with a higher frequency of UPF consumption is associated with a diet high in sugar, salt, and fat brought about by UPF consumption. In addition, studies are confirming that the increase in UPF consumption in addition to causing obesity, also leads to a series of metabolic diseases and chronic diseases in the body, leading to metabolic disorders in the body, affecting the body’s environment, leading to various types of chronic diseases, cardiovascular and cerebral vascular diseases, but also lead to the psychological symptoms of the important reasons for the increased (40, 41). However, the findings are not entirely consistent. It’s been found that dark chocolate and nuts, even though they are considered UPFs, are actually linked to a lower risk of developing cardiovascular disease. So, while they are sweet snacks that fall under the UPF umbrella, they have got some real health benefits going for them (42). In addition, it has been shown that dark chocolate, which is also in the UPF, is associated with a reduced risk of death and cardiovascular disease (43). This may also be an important reason why the joint effect of UPF consumption and duration of exercise analyzed by gender in the present study had no significant effect on psychological symptoms. The reason for this may be that the NOVA classification system is unable to fully encompass all the complexities of food processing, which can lead to misclassification of foods, resulting in inconsistent findings across studies. Then again, differences in the level of processing in different studies of the same food may also be an important reason for inconsistent results, such as differences in the amount of added sugar leading to different health benefits. The present study concludes that there are inconsistent and even widely differing findings on the effects of different products of UPF foods on adolescent health, and therefore future conclusions regarding the association between UPF consumption and psychological symptoms should be drawn with caution. Of course, we aim to correct this misperception and should not severely restrict all UPFs; after all, some UPFs are beneficial to health, such as dark chocolate or nuts.

The results of the present study also showed that the prevalence of psychological symptoms among Chinese adolescents was lower the longer the duration of exercise, which is consistent with the findings of many past studies (44, 45). Studies have shown that increased physical activity time improves blood circulation in the body, promotes increased blood flow to the brain, and promotes healthy brain development, which in turn has a positive effect on the development of mental health (46). Some research shows that working out can help balance your gut bacteria and reduce inflammation in the body, this can lead to better hormone levels, which is a big plus for keeping your mental health in check (47). The results of this study showed that the prevalence of psychological symptoms was 25.9% for those whose duration of exercise was <30 min/day, while a duration of exercise of 30 min/day or more could greatly reduce the prevalence of psychological symptoms to only 18.4–19.0%. This suggests that maintaining a duration of exercise of 30 min or more per day has a positive effect on adolescent mental health. Previous research has shown that when teens get active and exercise, their bodies go through some cool changes. Specifically, it cranks up the production of endorphins—often called “feel-good” hormones. These endorphins help keep their mood up and promote a positive mental state (48). In conclusion, most past studies have supported the positive effects of physical exercise on adolescent mental health, consistent with the findings of this study (49). However, while maintaining a positive duration of exercise there should also be a focus on keeping sports injuries from occurring. Excessive physical exercise leading to the occurrence of sports injuries can also affect the mental health of adolescents and hurt them (50). Therefore, it is especially important to maintain a reasonable duration of exercise.

The present study has certain strengths and limitations. In terms of strengths, Firstly, to the best of our knowledge, this study is the first to use a national sample from China to analyze the associations between adolescent UPF consumption, duration of exercise, and psychological symptoms, which provides some help for adolescent mental health development and intervention. Second, this study included several covariates to analyze the association between UPF consumption, duration of exercise, and psychological symptoms more objectively. In addition, the sample size of this study is large and has a certain representativeness and universality. However, this study also has some limitations. First, this study was a cross-sectional study that only analyzed the associations between UPF consumption, duration of exercise, with psychological symptoms, but not the causal relationship between them. Prospective cohort studies should be conducted in the future to analyze the causal relationship. Second, the questionnaire used in this study to evaluate UPF consumption and duration of exercise was affected by the individual’s ability to recall, which inevitably resulted in some discrepancies with the real situation. In the future, objective measurement instruments should be used for relevant assessments.

## 5 Conclusion

There is a relationship between UPF consumption and duration of exercise with psychological symptoms in Chinese adolescents. The higher the frequency of UPF consumption, the higher the prevalence of psychological symptoms, and the longer the duration of exercise, the lower the prevalence of psychological symptoms. The joint effect analysis of UPF consumption and duration of exercise did not reveal a significant association with psychological symptoms in boys and girls. The study suggests that in the future, adolescents should reduce UPF consumption and increase the duration of exercise to reduce the prevalence of psychological symptoms. Schools and education departments should take measures to limit UPF sales on campus and around schools, reduce UPF availability, and reduce UPF consumption. In addition, schools and education departments should strengthen supervision to ensure the duration of extracurricular exercise for 1 h a day.

## Data Availability

The raw data supporting the conclusions of this article will be made available by the authors, without undue reservation.
