# Supplementary material for: Associations between ultra-processed food consumption and duration of exercise with psychological symptoms in Chinese adolescents: a nationwide cross-sectional survey
Source: Front Nutr. 2025 Jun 17;12:1591909. doi: 10.3389/fnut.2025.1591909 (PMC12209267; doi:10.3389/fnut.2025.1591909)
Supplement: Supplementary file 1 [file Table_1.doc]

Supplementary Table 1 UPF classification

| Category | Example | Reason for Classification |
| --- | --- | --- |
| Beverages | Coca-Cola | Contains high fructose corn syrup, artificial flavors, and colors. Highly processed for consistency and shelf life. |
|  | Red Bull | Contains artificial sweeteners, caffeine, taurine, and B-vitamins. Processed for energy boost. |
|  | Gatorade | Contains artificial flavors, colors, and electrolytes. Processed to replace fluids and minerals. |
|  | Vitamin Water | Contains artificial flavors, sweeteners, and added vitamins. Processed for enhanced taste and health claims. |
|  | Pepsi | Contains artificial additives and undergoes significant processing to create a carbonated beverage. |
| Processed Meats | Hot Dogs | Made from reconstituted meat with added preservatives like sodium nitrite and artificial flavors. |
|  | Deli Ham | Contains added sodium, preservatives, and artificial flavors to enhance taste and extend shelf life. |
|  | Chicken McNuggets | Made from reconstituted chicken meat with added fillers, preservatives, and artificial flavors. |
| Baked Goods | Oreos | Contains refined sugars, artificial flavors, and preservatives. Processed for consistent texture and taste. |
|  | Snickers | Contains high amounts of sugar, artificial flavors, and preservatives. Processed for specific texture and taste. |
|  | Nutella | Contains added sugars, palm oil, and artificial flavors. Processed for smooth texture and sweet taste. |
| Convenience Foods | Instant Ramen | Contains refined wheat flour, artificial flavors, and preservatives. Processed for convenience and long shelf life. |
|  | Lasagna | Contains processed ingredients, artificial flavors, and preservatives. Designed to be convenient and have a long shelf life. |
|  | Noodle Soup | Contains artificial flavors, preservatives, and high amounts of sodium. Processed for long shelf life and consistent taste. |
| Snacks | Lays Classic Potato Chips | Contains refined potatoes, artificial flavors, and preservatives. Processed for specific texture and taste. |
|  | Granola Bars | Contains high amounts of sugar, artificial flavors, and preservatives. Processed for convenience and long shelf life. |
|  | Fruit Gushers | Contains artificial flavors, colors, and high amounts of sugar. Processed for chewy texture and sweet taste. |
| Breakfast Cereals | Frosted Flakes | Contains high amounts of sugar, artificial flavors, and preservatives. Processed for convenience and appeal to children. |
|  | Mini-Wheats | Contains refined grains, artificial flavors, and high amounts of sugar. Processed for specific texture and taste. |
| Desserts | Ice Cream | Contains artificial flavors, stabilizers, and high amounts of sugar. Processed for creamy texture and long shelf life. |
|  | Jell-O Pudding | Contains artificial flavors, colors, and preservatives. Processed for convenience and long shelf life. |
|  | Snack Cakes | Contains refined sugars, artificial flavors, and preservatives. Processed for convenience and long shelf life. |
